# Potential negative consequences of non-consented switch of inhaled medications and devices in asthma patients

**DOI:** 10.1111/ijcp.12202

**Published:** 2013-06-16

**Authors:** U S Björnsdóttir, S Gizurarson, U Sabale

**Affiliations:** 1 Department of Allergy and Immunology, University of IcelandReykjavik, Iceland; 2 Faculty of Pharmaceutical Sciences, University of IcelandReykjavik, Iceland; 3 Department of Health Economics, AstraZeneca NordicSE-151 85, Södertälje, Sweden

## Abstract

**Background**Asthma requires individually tailored and careful management to control and prevent symptoms and exacerbations. Selection of the most appropriate treatment is dependent on both the choice of drugs and inhaler device; however, financial pressures may result in patients being switched to alternative medications and devices in an attempt to reduce costs.

**Aim**This review aimed to examine the published literature in order to ascertain whether switching a patient’s asthma medications or device negatively impacts clinical and economic outcomes.

**Materials and methods**A literature search of MEDLINE (2001–13 September 2011) was conducted to identify English-language articles focused on the direct impact of switching medications and inhaler devices and switching from fixed-dose combination to monocomponent therapy via separate inhalers in patients with asthma; the indirect impacts of switching were also assessed.

**Results**Evidence showed that non-consented switching of medications and inhalers in patients with asthma can be associated with a range of negative outcomes, at both individual and organisational levels. Factors that reduce adherence may lead to compromised symptom control resulting in increased healthcare resource utilisation and poorer patient quality of life.

**Discussion**The consequences of a non-consented switch should be weighed carefully against arguments supporting an inhaler switch without the patient’s consent for non-medical/budgetary reasons, such as potential reductions in initial acquisition costs, which may be associated with subsequent additional healthcare needs.

**Conclusion**Given the increasing pressure for reduced costs and efficient allocation of limited healthcare resources, an additional investment in ensuring high medication adherence may lead to greater savings due to a potentially decreased demand for healthcare services. In contrast, savings achieved in acquisition costs may result in a greater net loss due to increased healthcare consumption caused by decreased asthma control.

**Review criteria**

A literature search of the MEDLINE database was conducted to identify English-language articles with content specific to the direct impact of switching medications and inhaler devices and switching from fixed-dose combination to monocomponent therapy via separate inhalers in patients with asthma; the indirect impacts of switching were also assessed. Conference abstracts were not included.

**Message for the clinic**

Switching medications and inhaler devices in patients with asthma without their consent or a medical need can result in increased demand for healthcare services. Prescribers should, therefore, not only consider the acquisition costs that may be potentially reduced by switching patients to lower cost products but also the potential subsequent costs of additional healthcare needs.

## Introduction

Asthma is a complex disease requiring careful and individually tailored treatment 1. The main management goal is to control and prevent symptoms and exacerbations to achieve optimal lung function and quality of life 2. Selection of the most appropriate treatment regimen is dependent on both the choice of drugs and inhaler device 3. This should be a collaborative process between the physician and patient as the behaviour of both is an important determinant of the level of asthma control achieved 4. Once an effective treatment strategy has been found, it is feasible that the patient may remain on that regimen for many years 5. However, as asthma is a common condition associated with a considerable burden on healthcare budgets, there is always pressure for cost reductions 6,7. Switching a patient from one product to another should be performed to improve the management of symptoms, and increase patient compliance and convenience. Nevertheless, when financial issues arise, a likely strategy could be to switch well-controlled patients without their consent to potentially reduce treatment costs.

**Figure 1:**
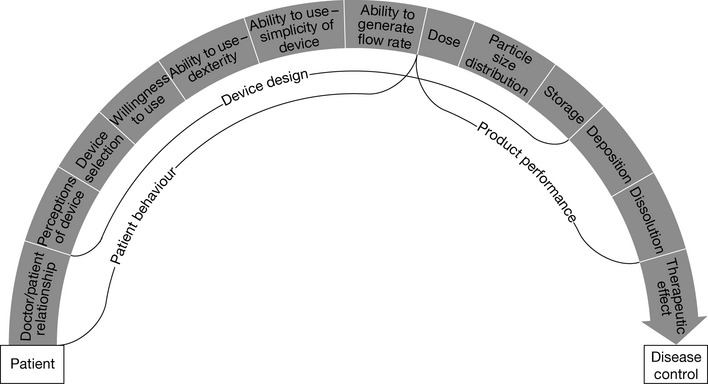
Stages between use of an inhaler by the patient and the therapeutic effect. Figure adapted from Price 7

There are a large variety of inhaler devices available, and differences in design, handling technique, durability (i.e. shelf-life) and price between branded and analogue products can be great. Successful management of asthma symptoms is dependent on a number of factors (Figure–1). One key factor is correct inhaler use 9,10. Patients are more likely to achieve better asthma control as a result of successful dose delivery when they become familiar with a particular device. In the case of switching, it is crucial that the reason for the switch has been properly explained to the patient and instructions for operating the device correctly have been clearly demonstrated 5. Unfortunately, this does not always occur and reports of asthma patients having their medications switched without their consent (non-consented switch) have emerged 5.

Non-consented switching means that patients do not receive any counselling from their healthcare provider about the new medication and device, which may result in poor inhalation technique. This can negatively impact adherence and asthma control, and evidence suggests that treating asthma medications as interchangeable on an interclass basis can have detrimental effects on patient outcomes 5,7. It is therefore possible that switching may not result in cost savings, because of increases in clinic visits for education and support and negative impacts on asthma control, resulting in higher short- and long-term healthcare costs.

### Objective

The objective of this review, which primarily focused on dry powder inhalers (DPIs), was to review the published literature to obtain a clearer picture regarding whether switching a patient’s asthma medications or device negatively impacts clinical and economic outcomes.

## Methods

Data were derived from published English-language papers listed in MEDLINE, and accessed via PubMed according to defined search terms. Searches were limited to full publications only; conference abstracts were not included. Only articles published between 2001 and 13 September 2011 were included. Three different searches were performed using keywords to identify publications that focused on:

- Monocomponents vs. fixed-dose combination (FDC) therapy: searches combined the terms bronchodilator and inhaled corticosteroids (ICS), asthma, single inhaler and combination inhaler.
- Consequences of switching devices: searches combined the terms bronchodilator and ICS, asthma, FDC or free-combination therapy, switching, device or inhaler (single inhaler or separate inhalers), adherence or compliance, asthma or symptom control and healthcare utilisation or costs.
- Adherence, cost-effectiveness and quality of life: searches combined the terms bronchodilator agents or ICS or glucocorticoid, adherence or compliance, cost-effectiveness or cost or health economics, quality of life, device or inhaler, training or technique or education or instruction, fixed or combination therapy or single inhaler therapy or separate inhaler or FDC or free combination or monotherapy or monocomponents and asthma. Resource use and resource utilisation were not included as search terms. This search was also limited to articles published between 2001 and 13 September 2011.

Abstracts of the articles identified via the three searches were analysed for content specific to the following topics:

- Direct impact of switching, including switching from combination therapy to monotherapy
  - Pharmaceutical performance
  - Inhalation technique
  - Adherence
  - Asthma control
- Indirect impact of switching
  - Healthcare resource consumption and costs.

## Results

### Direct impact of switching

The direct impact of switching has implications for pharmaceutical performance, inhalation technique and adherence, all factors that ultimately affect asthma control and quality of life 3–13.

#### Pharmaceutical performance

Currently, there are a large number of DPIs available; these devices differ greatly in their design and features 14. The effectiveness of devices with regard to the delivery of inhaled medications is dependent on many factors, such as design characteristics, medication, carrier, particle size, shape and density, lung deposition and ease of use 9. The increase in supply of DPIs has not correlated with a growth in knowledge of the disparity between products in terms of lung deposition and dose-delivery capabilities; there appears to be a prevailing assumption that devices are interchangeable and deliver the same dose of medication regardless of the inhaler 14. Differences between dose-delivery capabilities of DPIs have been explored in several studies, demonstrating that the pharmaceutical performance of different devices delivering the same molecule can vary widely, which has a great impact on asthma outcomes. Devices should, therefore, not be considered to be interchangeable with regard to pharmaceutical performance 12.

#### Inhalation technique

Dry powder inhalers also differ with respect to inhalation technique. Correct technique is critical for delivering the correct drug dose to the airways, and patient education is crucial 9–15. Each device requires a certain level of inhalation flow to ensure efficient disaggregation of the formulation; this may be problematic, for example, in children and the elderly, who may unable to produce the required inhalation flow, resulting in medication underuse.

Successful switching requires instruction on the correct inhaler technique and regular monitoring; incorrect use of a device is one of the most frequent concerns patients express after switching 9–17. To prevent confusion regarding techniques and the introduction of critical handling errors (i.e. those that result in little or no delivery of medication to the lungs), different types of inhalers should not be mixed for an individual patient 9–19.

When a patient follows an instruction leaflet, the first attempt to use a new device is often unsuccessful with a high probability of handling errors 15. This indicates that patients find it hard to understand written instructions on inhalation technique and need assistance and careful monitoring by a healthcare specialist. Reduced health literacy levels have been associated with poorer adherence in patients with respiratory diseases, and can be particularly relevant to certain subgroups of patients, for example, elderly people who may have difficulty reading 20. Elderly patients may also have problems recalling the correct technique, even if it has been demonstrated by a healthcare professional. In addition, depression has been identified as a risk factor for non-adherence, and such patients may be particularly sensitive to switching 21. The need for additional physician visits was underlined by Schulte et–al. who noted that patients were better at handling the device after listening to instructions from a physician than after independently reading a leaflet 15. Indeed, in a recent cross-sectional observational study, lack of instruction on correct inhaler technique was the only modifiable factor significantly associated with critical handling errors 22.

#### Adherence

Adherence to treatment is essential for optimal asthma control; however, studies indicate that patients are poorly adherent and generally underuse their asthma medications 13,17. Decreased adherence, whether intentional or unintentional, is a common outcome of switching 17. Unintentional non-adherence may occur because of individual constraints, such as poor handling technique, critical handling errors, an inability to recall consultations, or environmental constraints, such as costs or difficulties accessing prescriptions. Intentional non-adherence may arise if a patient has a perception of asthma or a particular belief making them disinclined to adhere to treatment; for example, concerns regarding ICS or if a patient has low motivation to use an inhaler that is not their device of choice 4,15.

It is important that prescribers consider patient needs and preferences when choosing the most appropriate treatment choice as these factors contribute greatly to adherence 25–26. The choice is highly dependent on individual patient- and device-related factors such as availability of the drug and dose in the specific device, ability to develop and maintain an effective technique, suitability of the device, the fit of the regimen to the person’s lifestyle, preference for and willingness to use a particular device, and patient confidence in the safety and efficacy of the treatment 25,26. These stages between inhaler use and therapeutic effect are outlined in Figure–1.

Adherence is also dependent on several non-drug- or -device-related factors, including patient–physician partnership and level of patient satisfaction with their device 25–26. Patients do not have equal preference for different DPIs 15, and for the majority of patients, the higher the level of device satisfaction, the greater the likelihood of adherence leading to better outcomes 25.

Few studies have directly assessed adherence to FDCs of ICS/long-acting β_2_-agonists (LABA) compared with ICS plus LABA administered via separate inhalers, as a true double-dummy technique is not possible. However, data from an open-label, randomised study and two retrospective observational analyses show that adherence tends to be higher with FDCs administered via a single inhaler rather than monocomponents via separate inhalers 28,29. Data also suggest that the minor benefits in certain patient-reported outcomes associated with FDCs 31, as well as fewer withdrawals from treatment and fewer prescriptions for other asthma medications, may be the result of single-inhaler therapy being more convenient than separate inhalers 32,33. In a real-world setting, this is likely to translate into greater patient adherence 32.

The consequences of a non-consented inhaler switch were assessed qualitatively by Doyle et–al. via semi-structured face-to-face interviews with patients (*n*–=–19) 5. Patients described struggling to actuate the new device, overuse of medication (especially rescue medication), feeling disempowered and a lack of personal control over their medical condition, damaging the relationship with their doctor as a consequence. The study concluded that the negative impact of switching must be seriously considered.

Important themes associated with interchangeability of DPIs were identified in a survey of asthma patients or parents of children aged 5–14–years with asthma (*n*–=–499) from Australia, Canada, France, Germany and the UK using a Delphi process 35. Here, the majority (83%) of patients would raise concerns and questions regarding the switch, while one-half (51%) would oppose switching. The majority of patients (61%) also thought that it would be confusing to have their device changed, while 23% would ask for more information about the change or training regarding the new device.

#### Asthma control

Reduced medication adherence is directly related to reduced asthma control 36,37, resulting in an increased frequency of exacerbations 37–38 and asthma-related mortality 39. Retrospective analysis of a managed-care database examined the association between adherence and exacerbations in asthmatic patients with prescriptions for controller medications (*n*–=–97,743) 38. More adherent patients were significantly less likely to experience exacerbations than less adherent patients. For all adherence cut-off points (≥–2 through ≥–6 prescriptions), there were significantly fewer exacerbations in more adherent than less adherent patients after adjusting for covariates. As the criteria for adherence became more stringent, more adherent patients became increasingly less likely to have an exacerbation than less adherent patients. Similarly, adherence to ICS has been shown to be significantly and negatively correlated with the number of emergency department visits, number of prescription fills of an OCS and total days of OCS use 37.

The increased probability of handling errors resulting from unfamiliar inhalation techniques may also result in reduced asthma control as a result of under-dosing 9,16. The causal relationship between switching and lower asthma control was assessed by Thomas and Williams 40. Patients who were switched without being consulted by a physician had worse asthma control than those who stayed on the same treatment. The switched cohort were also more likely to use SABA, a marker for decreased asthma control, using 0.38–extra doses per day of SABA compared with the control group (p–<–0.001). After adjusting for baseline confounding factors, the overall likelihood of unsuccessful treatment among the switched cohort was substantially higher than for those who stayed on the same medications (odds ratio: 1.92; 95% confidence interval: 1.47, 2.56; p–<–0.001).

The causal relationship between poor device handling and poor asthma control has been investigated in two studies 22–36. In the study conducted by Molimard and Le Gros 36, the Asthma Control Score (ACS; where entirely controlled asthma was indexed by 0 and uncontrolled by 9) was calculated from data recorded in routine consultations of 4362 patients with persistent asthma using maintenance ICS-only treatment and correlated with patient characteristics, compliance (using two methods), and critical inhaler handling errors. More than 20% of patients were using their inhalers incorrectly, which was associated with a 0.84-point increase in the ACS. ACS was substantially better in patients who missed ≤–4 doses per week than in those who were poorly compliant (missed >–4 doses per week). Asthma control was inadequate in 63% of those who missed >–4 doses per week compared with 38% of those who missed ≤–4 doses per week. Similarly, Melani et–al. investigated the relationship between inhaler device handling and disease control in 1664 adults, 42% of whom had asthma. Inhaler misuse was associated with an increased risk of poor disease control as measured by the Asthma Control Test (p–<–0.001) 22.

Non-adherence leads to numerous adverse events associated with uncontrolled asthma 37, which, in turn, result in increased demand for healthcare resource 8. Therefore, it is necessary to emphasise the importance of weighing the possible cost of adverse events against acquisition cost savings before initiating mass switching.

### Indirect impact of switching

#### Healthcare resource consumption and costs

**Figure 2:**
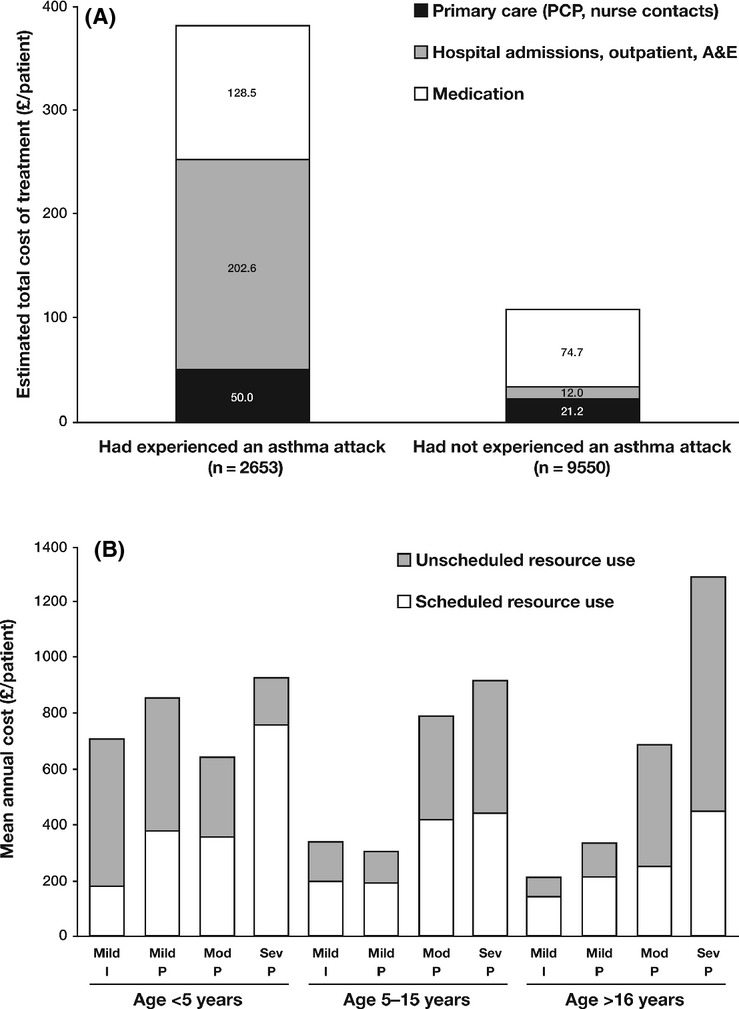
Costs of managing asthma patients: (A) with or without asthma exacerbations (published data on unit costs were used to assess the cost pattern of healthcare for each patient) 42, figure adapted from Price 7; and (B) with scheduled and unscheduled asthma healthcare visits by symptom severity and age group, figure adapted from Williams et–al. 44. A&E, accidence and emergency; I, intermittent; Mod, moderate; P, persistent; PCP, primary care physician; Sev, severe

Non-consented switching and switching for non-medical reasons can have direct negative effects on disease control, resulting in increased healthcare resource consumption and costs 8. Indeed, poorly controlled asthma accounts for almost 50% of the total asthma healthcare burden 41. Annual treatment costs incurred by patients who have an exacerbation are three times higher than in those who do not (Figure–2A) 42. In addition, costs of asthma exacerbations in secondary care increase with increasing severity of exacerbations 43.

The difference in costs of scheduled and unscheduled asthma healthcare visits in Europe was estimated by Williams et–al. 44. Unscheduled visits accounted for almost one-half of the total asthma management healthcare expenditure (Figure–2B). Different patterns of unscheduled healthcare consumption were observed according to asthma severity. Among adults, 11% of patients reported at least one emergency room visit (ranging from 6% with mild intermittent symptoms to 17% with severe persistent symptoms). If unscheduled physician visits are considered, 24% of patients had at least one unscheduled visit (ranging from 15% to 38%, depending on disease severity).

**Figure 3:**
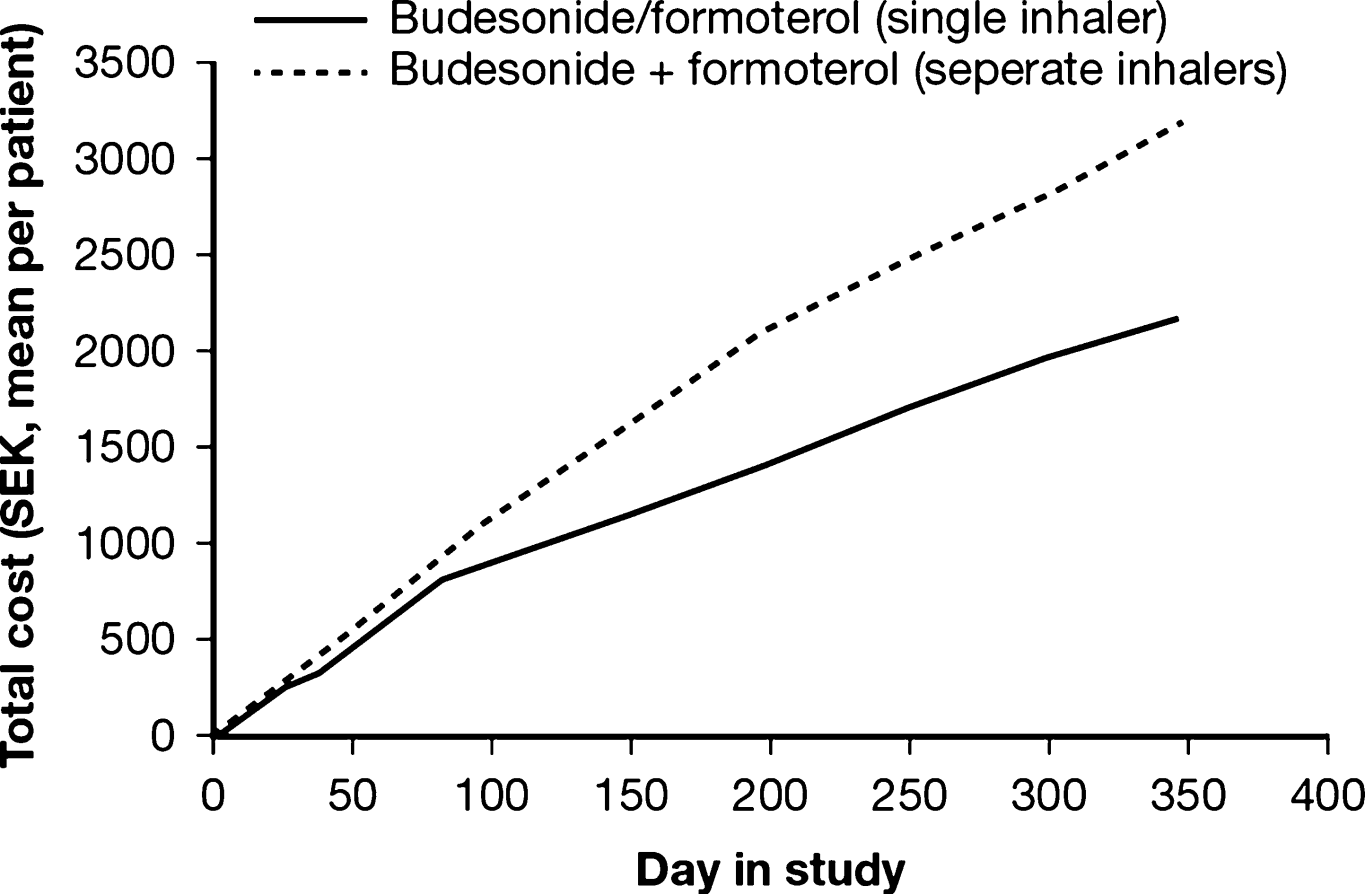
Change in direct and indirect total costs of fixed-dose budesonide/formoterol therapy vs. monocomponent therapy (1999 cost year) in patients with asthma, excluding study medication costs, over time; figure reproduced from Rosenhall et–al. 33. As of April 2013: 1 SEK–=–0.10 GBP–=–0.53 USD

In other chronic diseases, FDC therapies have been shown to improve adherence compared with individual monocomponents, thereby improving disease control 45–46; this is also thought to be applicable in asthma 32,33. In Sweden, combination budesonide (BUD)/formoterol (FM) inhalers were significantly more cost-effective than using separate BUD and FM inhalers 33. The cost of study medication was lower for the FDC BUD/FM inhaler than for separate BUD and FM [7822 Swedish krona (SEK) per year vs. 8530 SEK per year, respectively], and direct and indirect medical costs associated with treatment were considerably lower for the combination-inhaler group compared with separate-inhaler group (Figure–3).

## Conclusion

**Figure 4:**
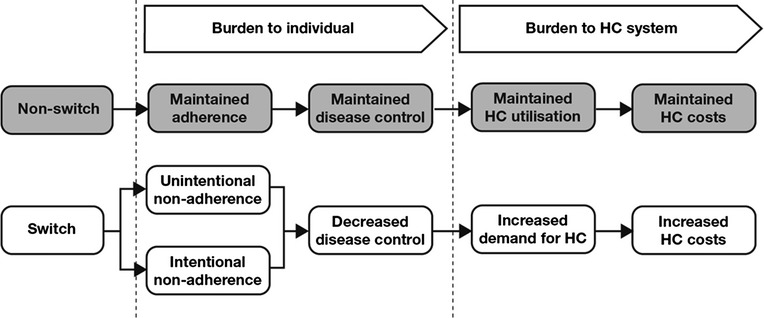
Potential clinical and economic consequences of switching well-controlled patients with asthma for cost reasons. HC, healthcare

The causal chain of events discussed above, and outlined in Figure–4, provides us with an opportunity to understand the complexity of possible negative outcomes associated with switching asthma medications and inhaler devices, driven by non-patient-related factors, at individual and organisational levels. A patient who is uncomfortable with handling an inhaler is at greater risk of critical handling errors, which compromises asthma control. Worsened asthma symptoms and an increased need for additional consultations regarding device handling technique logically lead to increased demand for healthcare services. Subsequently, higher healthcare service consumption as a result of increases in physician hours and other healthcare resource allocation results in higher costs.

To conclude, given the continually increasing pressure for reduced costs and efficient allocation of limited healthcare resources, an additional investment in ensuring high medication adherence may lead to greater savings because of potentially decreased demand for healthcare services. In contrast, savings achieved in acquisition costs may result in a greater net loss because of increased healthcare consumption, caused by decreased asthma control.
